# Prevention and public health approaches to trauma and traumatic stress: a rationale and a call to action

**DOI:** 10.3402/ejpt.v7.29715

**Published:** 2016-03-18

**Authors:** Kathryn M. Magruder, Nancy Kassam-Adams, Siri Thoresen, Miranda Olff

**Affiliations:** 1 Department of Psychiatry & Behavioral Sciences, Military Science Division, Medical University of South Carolina, Charleston, SC, USA; 2 Department of Public Health Sciences, Division of Epidemiology, Medical University of South Carolina, Charleston, SC, USA; 3 Center for Injury Research and Prevention, Children's Hospital of Philadelphia, Philadelphia, PA, USA; 4 Norwegian Centre for Violence and Traumatic Stress Studies, Oslo, Norway; 5 Department of Psychiatry, Academic Medical Center, University of Amsterdam, Amsterdam, The Netherlands; 6 Arq Psychotrauma Expert Group, Diemen, The Netherlands

**Keywords:** Posttraumatic stress disorder, trauma, prevention, public health

## Abstract

**Background:**

The field of trauma and traumatic stress is dominated by studies on treatments for those who experience adversity from traumatic experiences. While this is important, we should not neglect the opportunity to consider trauma in a public health perspective. Such a perspective will help to develop prevention approaches as well as extend the reach of early interventions and treatments. The purpose of this paper is to provide an introduction to a public health approach to trauma and traumatic stress and identify key opportunities for trauma professionals and our professional societies (such as the International Society for Traumatic Stress Studies [ISTSS] and the European Society for Traumatic Stress Studies [ESTSS]) to increase our societal impact by adopting such an approach.

**Method:**

This paper reviews and summarizes key findings related to the public health impact of trauma. The special case of children is explored, and a case example of the Norwegian terrorist attacks in 2011 illustrates the potential for improving our response to community level traumatic events. We also discuss how professional organizations such as ESTSS and ISTSS, as well as individual trauma professionals, can and should play an important role in promoting a public health approach.

**Results:**

Trauma is pervasive throughout the world and has negative impacts at the personal, family, community, and societal levels. A public health perspective may help to develop prevention approaches at all of these levels, as well as extend the reach of early interventions and treatments.

**Conclusions:**

Professional organizations such as ESTSS and ISTSS can and should play an important role in promoting a public health approach. They should promote the inclusion of trauma in the global public health agenda and include public health in their activities.

The field of trauma and traumatic stress is dominated by studies on treatments for individuals who experience adversity from traumatic experiences. While effective individual treatment is vitally important, we should not neglect the opportunity to consider trauma in a public health perspective. Such a perspective will help to develop prevention approaches as well as extend the reach of early interventions and treatments. In this paper, we provide an introduction to a public health approach to trauma and traumatic stress and identify key opportunities for trauma professionals and our professional societies (such as the International Society for Traumatic Stress Studies [ISTSS] and the European Society for Traumatic Stress Studies [ESTSS]) to increase our societal impact by adopting such an approach. A recent panel, organized by ISTSS and presented at the 2015 ESTSS conference in Vilnius, Lithuania, addressed prevention and public health approaches to trauma and traumatic stress. Based on that panel, the current paper summarizes key findings of a recently completed report of the ISTSS Task Force on Trauma and Public Health and discusses implications for research, practice, and policy. The special case of children is explored, and a case example of the Norwegian terrorist attacks in 2011 illustrates the potential for improving our response to community level traumatic events. We also discuss how professional organizations such as ESTSS and ISTSS, as well as individual trauma professionals, can and should play an important role in promoting a public health approach.

## Public health approach

In this section, we provide an introduction to several key concepts and argue for their inclusion in the research, practice, and policy agenda of the trauma field: the public health impact of trauma, the prevention model, current progress and challenges in designing and implementing trauma-informed services, secondary prevention, and early intervention for individuals and communities exposed to a range of types of trauma.

### Public health impact of trauma

Trauma is highly prevalent throughout the world. Data from the WHO World Mental Health Surveys of adults show that at some time in their life, 9% of all respondents had experienced collective violence, 17% had experienced interpersonal violence, 26% had witnessed violence, 23% had experienced sexual or partner violence, 36% accidents or injuries, and 41% other types of trauma (Kessler & Üstün, 2008). Considering all traumas, 70% of all respondents had experienced at least one type.

There are, however, some variations by country (although it is possible that some variations reflect differences in willingness to report or some other aspects of measurement). Among countries surveyed, Bulgaria had the lowest prevalence of trauma at 29% and Ukraine had the highest at 84% (Kessler & Üstün, 2008). Only one other country (Romania at 42%) was below 50%.

There are also variations in trauma exposure by other characteristics. For example, women are more likely to experience sexual assault and intimate partner violence, while men are more likely to experience physical assaults, threats with a weapon, and combat exposure (e.g., Olff, Langeland, Draijer, & Gersons, 2007; Sprague & Olff, 2014). Young children are at high risk of physical abuse by a caregiver, witnessing domestic violence, and kidnapping. Racial and ethnic minorities are also more often targets of violence as are those of gay, lesbian, or bisexual orientation (Roberts, Austin, Corliss, Vandermorris, & Koenen, 2010). Of note, living in a community of lower socioeconomic status is also a risk factor for trauma exposure (Nakagawa & Shaw, 2004).

Trauma exposure has particularly pernicious effects on both mental and physical health (Qureshi et al., 2010; Qureshi, Pyne, Magruder, Schulz, & Kunik, 2009), even decades later (Goldberg et al., 2014; Magruder et al., 2015). Those exposed are at elevated risk of major depression, anxiety disorders, substance abuse and dependence, and behavior disorders in children and adolescents (Breslau, Davis, Peterson, & Schultz, 2000; Breslau, Davis, & Schultz, 2003; McLaughlin et al., 2012). As the number of lifetime traumatic events increases, so too does the likelihood of developing a chronic medical condition (Scott et al., 2013).

Trauma is problematic not only for individuals but also for communities (Ajduković, 2013). Disasters, terrorism, and armed conflicts can undermine the social fabric of communities and erode the public health infrastructure and systems that are most needed following community-level trauma. All too often, there is forced migration (Turner, 2015) or even voluntary migration (as is seen in the current exodus of refugees from the Middle East) impelled by the dangerous and untenable living conditions in home countries. Other community impacts of trauma include dissolution of support networks, disruptions in the provision of social services, reduced access to health and mental health treatments, erosion of social capital, lost productivity, and high societal costs (Kawachi & Subramanian, 2006; Zwillich, 2006).

### The public health model and opportunities for prevention

We believe that trauma professionals whose primary training is grounded in individually oriented biopsychosocial models of trauma would benefit from adding a public health “lens” to their repertoire. The following presents several basic concepts that are key to the public health model as it applies to trauma.

**Fig. 1:**
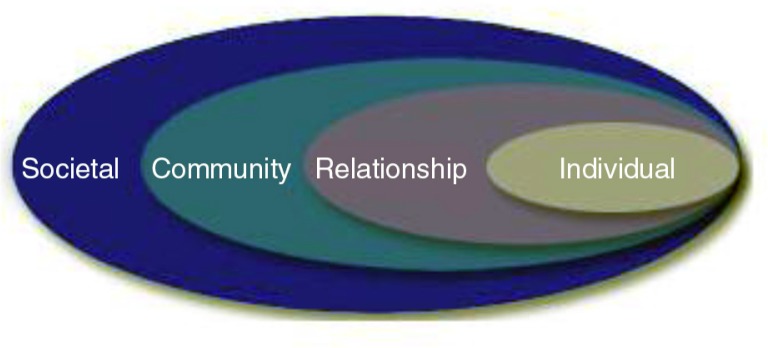
The social–ecological model, a framework for prevention from the US Centers for Disease Control and Prevention. (From Dahlberg & Krug, 2002).

In the field of public health, early models were relatively straightforward, dealing with understanding and eradicating infectious diseases with a single causal agent. These models have become more complex as public health scholars and practitioners attempt to address chronic diseases—including mental disorders—which are often multi-causal. The US Centers for Disease Control and Prevention have espoused a social–ecological model as a framework for prevention (see Fig. 1) (Dahlberg & Krug, 2002). This model emphasizes risk factors at multiple levels. At its core are the *individual* and his or her personal characteristics and factors. At the next level are *relationship* factors, which include family, marital, peer, and other interpersonal relationships. *Community* level risks include the immediate characteristics of a neighborhood, such as poverty level and safety. At the *societal* level, characteristics such as the norms and tolerance of a society for certain problems or certain issues may introduce additional risk or protective factors.

**Fig. 2:**
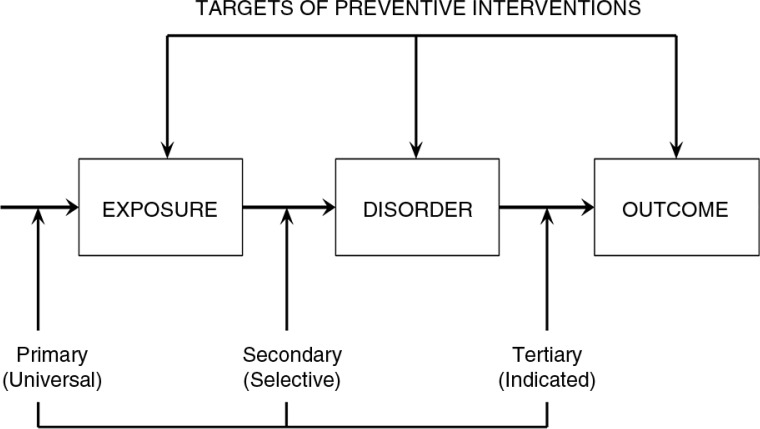
Targets of the three major classes of preventive interventions: primary, secondary, and tertiary. Adapted from Costello and Angold (1995).

Most importantly, by targeting risk factors at each of the levels, the social–ecological model points to a number of strategies for prevention and early intervention (see Fig. 2). Applying a framework that differentiates among *primary* prevention (preventing the actual occurrence of disease or illness), *secondary* prevention (intervening early in the disease process for cure or optimal outcomes), and *tertiary* prevention (preventing disability that often accompanies an illness or disease), there are numerous opportunities at the various levels to implement preventive interventions. While primary, secondary, and tertiary prevention stem from the public health framework, the terms universal, selective, and indicated prevention (Gordon, 1983) have later been proposed for targeting the population the intervention addressed. In this paper, we will use the public health terminology.

Opportunities for *primary prevention* at the individual level may include educating university students about the risk of sexual assault as alcohol consumption increases. At the relationship level, primary prevention aimed at school children could promote respectful peer relationships in order to prevent bullying. Community primary prevention efforts may call for better lighting in streets and pedestrian areas or establishing neighborhood-watch programs to prevent assaults and other forms of violence. Exciting efforts are underway to develop approaches that improve preparedness and resilience for high-risk communities in order to prevent the mental health consequences of trauma (Laborde, Magruder, Caye, & Parrish, 2013). At the societal level, policies that restrict possession of firearms, such as recent Australian firearm laws, may play a role in reducing rates of homicide, murder, and even suicide.

*Secondary prevention* targets posttrauma opportunities. At the individual level, these may include approaches such as psychological support for disaster survivors. At the relationship level, this includes intervention of couples in cases of domestic violence. Community-level preventive approaches include vigils in support of survivors and families following disaster or mass violence. At the societal level, policies that deny firearm possession for those convicted of domestic violence play an important role in preventing escalating violence.

At all levels, because a traumatic event is an identifiable point of onset, this provides opportunities for early preventive (secondary) interventions. Although research on prevention is lagging behind curative intervention studies, there are interesting developments in the area of early interventions ranging from e-health to neurobiology (Olff, Van Zuiden, & Bakker, 2015). For instance, approximately 20% of traumatized emergency department (ED) admitted individuals develop adverse mental health outcomes, including posttraumatic stress disorder (PTSD), depression, and anxiety, and screening shortly after trauma may help to identify individuals at high risk for adverse outcomes and target preventive (online) interventions selectively (Mouthaan, Sijbrandij, Reitsma, Gersons, & Olff, 2014; Sijbrandij et al., 2013). Although literature is scarce, several studies have shown that with a short early intervention, prevention of adverse outcomes is possible (Brunet, Bousquet Des Groseilliers, Cordova, & Ruzek, 2013; Mouthaan et al., 2013; Rothbaum et al., 2012). As one example, building on results from a randomized intervention that demonstrated decreases in alcohol consumption, injury recurrence, hospital readmissions, and motor vehicle violations (Gentilello et al., 1999), in 2007 the American College of Surgeons Committee on Trauma required all Level I trauma centers in the United States to conduct screening and brief intervention for alcohol problems. In turn, this policy resulted in a 2.2% decrease in the probability of readmission (Hinde, Bray, Aldridge, & Zarkin, 2015). This policy demonstrates the indirect benefits of alcohol screening and brief intervention for trauma prevention.

E-health, and in particular mobile apps, forms an exciting new public health approach (Olff, 2015). Embedding trauma-informed approaches into a wide variety of service systems that interact with trauma-exposed adults or children can also support secondary prevention of trauma sequelae and efficiently extend the reach of interventions.

As *tertiary prevention* is aimed at preventing disability and complications of illness, at both the individual and relationship levels, traditional treatment strategies are good examples. Clinical interventions are intended to relieve symptoms and distress, as well as prevent the development of comorbidities. In primary care settings, up to half of the individuals who meet criteria for PTSD may go unrecognized (Magruder et al., 2005); thus, implementing screening programs accompanied by training primary care providers in brief interventions and referral can serve to improve the reach of extant interventions. Furthermore, adapting proven interventions for delivery via e-health and mobile apps also has good potential to extend reach at reduced cost. Similarly, couples therapy is designed to treat dysfunctional relationships and prevent the progression of further problems. At the community level, programs that promote support and acceptance of stress-related conditions may help with accommodations that address disabilities and encourage trauma survivors to seek treatment. At the societal level, peace-keeping efforts would clearly go a long way to reduce re-traumatization.

### Public health policy

Organizations such as ISTSS and ESTSS have an important role to play in informing policy makers of the scientific and clinical evidence regarding trauma and its impact, and in advocating for public health policies relevant to the impact of trauma for individuals, families, communities, and nations. Public health policies can help to shape societal norms and are ideally informed by an evidence base. In addition, they can help to extend services to more of those in need and reduce societal cost. Examples of successful public health policy efforts that have led to improvements in population-level health include tobacco control, motor vehicle safety, and prevention and control of infectious diseases.

In the area of trauma, such policies should focus on the prevention of traumatic events, extending the reach of early intervention services for survivor communities, and reducing the stigma that often accompanies trauma victimization. Prevention strategies not only serve the individual but may also reduce costs for society. If not prevented or timely and adequately treated, the course of traumatic stress consequences is generally chronic, severely impacting well-being and daily-life functioning (Goldberg et al., 2014). The economic burden associated with adverse mental health outcomes of trauma is substantial, encompassing both direct (health care utilization and medication) and indirect costs (lost productivity).

## A public health approach to addressing child trauma exposure

As one example of a compelling area of need where trauma professionals and organizations can make a difference, we consider a public health approach to addressing child trauma exposure. The vast population-level impact of trauma exposure among children and youth provides a compelling example of the need for a public health approach. Child trauma is very common, affecting millions of children and adolescents. Globally, 400 million children are injured each year and more than 70 million children each year are exposed to disaster (Guha-Sapir, Hoyois, & Below, 2014). International population surveys of schoolchildren indicate that a significant proportion experience violence from parents, peers, and strangers (United Nations Children's Fund, 2014). Recent meta-analyses examining international data from hundreds of studies substantiate the ongoing exposure of children and adolescents to physical and sexual abuse, across nations, continents, and cultures (Stoltenborgh, Bakermans-Kranenburg, Van Ijzendoorn, & Alink, 2013; Stoltenborgh, Van Ijzendoorn, Euser, & Bakermans-Kranenburg, 2011).

In addition, results from the Adverse Childhood Experiences (ACEs) Study indicate that a range of adverse childhood experiences have long-range health implications in adulthood. This includes, for example, elevated risk in adulthood for alcoholism, drug abuse, depression, suicide attempts, smoking, poor self-rated health, obesity, ischemic heart disease, cancer, skeletal fractures, liver disease, and chronic obstructive pulmonary disease (Anda et al., 2008; Felitti et al., 1998).

There are numerous opportunities at the community and societal levels for primary prevention of childhood injury, of children's direct and indirect exposure to violence, of sexual and physical abuse of children, and of disasters caused by humans. In terms of tertiary prevention of long-term sequelae of trauma, there are a number of excellent evidence-based treatments for posttraumatic stress and related mental health consequences in children. However, in many communities, mental health service systems are non-existent or over-burdened and globally, the existing mental health workforce is not adequately prepared to provide trauma-focused services for children and adolescents and their families. An additional barrier is that few trauma-exposed children and families seek out mental health services.

Thus, a public health approach that focuses on secondary prevention of the impact of trauma for children and adolescents is sorely needed. The collaboration between the WHO and US National Centers for Chronic Disease Prevention and Health Promotion (CDC), built around findings from the ACEs Study, is a good start and includes prevention of both the immediate consequences of childhood adverse experiences as well as long-term sequelae (Anda, Butchart, Felitti, & Brown, 2010). There is evidence that social support from family and classmates may help to prevent some of the long-term negative consequences of bullying and abuse in adolescence (Strøm, Thoresen, Wentzel-Larsen, Sagatun, and Dyb, 2014). Two key questions include: 1) Who are the children who need help to address the impact of trauma exposure and prevent the development of traumatic stress and related sequelae? 2) How and where might we find and serve these children in ways that help to reduce trauma's impact? Such services need not be delivered formally, or within traditional mental health service systems. This concept is congruent with recent calls to “reboot” mental health intervention research and practice more generally in order to address the population-level burden of mental health concerns (Kazdin & Blase, 2011). Rather than seeking to develop only interventions that are highly efficacious (if hard to deliver), a public health approach suggests balancing efficacy with “reach”—the proportion of the target population who will be able to be served and assisted by a given intervention (Kazdin & Blase, 2011; Koepsell, Zatzick, & Rivara, 2011). In this approach, researchers and practitioners ask not only “Is this intervention effective in addressing the impact of child trauma for individual participants/recipients?” but also “How far does it go to address the population-level burden of child trauma for children, adolescents, and families?” Two models that exemplify attention to population-level reach and that have begun to be applied to secondary prevention efforts to address child trauma are e-health approaches and trauma-informed service systems.

Several e-health interventions that address child trauma have been developed and evaluated (Cox & Kenardy, 2010; Kassam-Adams et al., 2016; Marsac, Kassam-Adams, Hildenbrand, Kohser, & Winston, 2011; Price et al., 2014; Ruggiero et al., 2015), and this is a promising area of research and practice. Mobile applications, in particular, hold the promise of wide reach (at reduced cost) because an increasing proportion of the world's population (including young people) have access to the Internet via mobile devices (Olff, 2015). The potential promise of e-health tools to efficiently reach huge numbers of individuals across language and national barriers is exemplified in a recent study of an online smoking cessation intervention for adults (Muñoz et al., 2015). In the model of “massive open online courses” (MOOCs) that regularly reach tens of thousands of learners internationally, this “massive open online intervention” reached nearly 300,000 individuals from 168 countries across a 30-month period at a low cost per visitor, was presented in Spanish and English, and was entirely self-guided. Success rates (quit smoking rates) were comparable with those observed in in-person interventions. A public health approach to child trauma might envision a massive open online intervention that reaches children and parents worldwide.

“Trauma-informed service systems” have been defined as systems that: 1) are aware of the impact of trauma for the individuals and families they interact with, 2) recognize the signs and symptoms of trauma exposure and trauma responses in children, families, and their own staff, 3) integrate this knowledge into programs and practices, and 4) take steps to avoid re-traumatizing those they serve (Substance Abuse and Mental Health Services Administration, 2014). In addition to the mental health and social service systems, many other systems regularly come into contact with trauma-exposed children, often quite close to the time of the child's exposure to a potentially traumatic event. These include schools, the health-care system, law enforcement (police and courts), as well as child protection or child welfare systems. There is a key role for professionals and staff in these systems, as they interact with children and families, to help reduce or mitigate the impact of trauma exposure. It is important to identify a compelling rationale for each system regarding the benefits of a trauma-informed approach for the outcomes that the system values (e.g., trauma's impact on academic performance for school systems, trauma's impact on physical health outcomes for health-care systems, trauma's impact on recidivism for law enforcement). It is also important to identify how professionals and staff in each system can utilize an awareness of trauma within the scope of their job and training, for example, a classroom teacher might modify the way he or she handles classroom disruptions to take into account a student's “triggers” related to trauma exposure; a pediatric nurse might provide specific assistance to help a parent remain present and supportive during a child's painful or distressing medical procedure; or a police officer might be sure to kneel down and address a child directly to explain what is happening when she must arrest the child's parent for domestic violence.

A public health approach is useful in 1) looking beyond the individual level to clarify the vast population impact of child trauma and 2) identifying a range of potential models to address this impact that go beyond individual interventions. The challenge to the trauma and public health fields is to address this population-level burden of child trauma. Balancing efficacy with reach, promising methods include (but are certainly not limited to) online and e-health tools, and embedding trauma-informed services within the many systems that comes into contact with children after trauma.

## The impact of the Norway 2011 terrorist attacks in public health perspective

The public health social–ecological model (Dahlberg & Krug, 2002) draws our attention to the various levels that may be targeted for prevention efforts and also for research. Most research on trauma and violence exposure, disasters, and terrorist attacks have so far focused on the individual level, for example, by investigating the relationship between individual exposure to trauma and later mental health. Individual perceptions of available social support have sometimes been included, but other ecological aspects are largely ignored. Characteristics of the individual's social network and the community and society in which the person is embedded may be key to effective prevention. As one example of the importance and usefulness of thinking across multiple levels in a social–ecological model, we report here on efforts by trauma professionals to apply this public health perspective in responding to the terrorist attacks in Norway in 2011.

Terrorist attacks differ in several ways from other traumatic events such as sexual assault, traumatic loss, physical violence, and car accidents. Terrorism aims to shock the public and to get media attention, thereby achieving political aims (Hoffman, 1999). This was the case with the attacks in Norway on July 22, 2011. The terrorist was quoted in Norway's largest newspaper: “The operation was just a formality… (I) had a pragmatic approach, would kill enough so that the manifesto would reach the world press” (Foss & Østli, 2011, author's translation). The target of a terrorist thus goes beyond the directly affected victims and aims at political and attitudinal change in society. The social–ecological model may thus be particularly well suited for research and prevention efforts following terrorist attacks.

In the case of the 2011 Norway attacks, *the individual level* constituted three groups: the survivors from the bombing at the Governmental quarter, the young people who were present at Utøya Island at the time of the shooting, and the bereaved families. We will concentrate here on the survivors from the shooting at Utøya Island. In most cases of terror and disasters, researchers will face sampling problems, but this was not the case with Utøya because the island posed a geographically isolated unit. The team responsible for planning the research and clinical response for the survivors therefore decided early on to conduct face-to-face interviews, to ensure that the research interview could also function as a security net, that is, individuals in need could be assisted in getting health services. In the first wave of assessments, 4–5 months after the attack, posttraumatic stress levels were more than six times higher in survivors than in the general population. This extremely high symptom severity may be related to the very severe trauma exposure experienced by the Utøya survivors.

*The relationship level* included parents, siblings, friends, colleagues, and others who were close to the Utøya survivors. Research after the Utøya attacks included the survivors’ parents. Parents’ symptoms and coping were studied in their own right, and parental mental health and coping also constituted a relationship level factor for the study of the survivors. Although recent years have seen shootings and terrorist attacks specifically targeting young people, such as the Beslan school hostage crisis, the Peshawar school attack in Pakistan, several school shootings in the USA and Finland, and the 2011 shooting attack at Utøya, little is known about parents’ health following their children's exposure to trauma. Results from the Utøya study show that parents of severely traumatized young people constitute a vulnerable group and should be targeted in early intervention and follow-up strategies.

*The community level* in this case included schools, colleges, universities, workplaces, and municipal health services. Municipal health services were mobilized for outreach services to individual survivors and their families and were also instructed to perform early screening. Unfortunately, factors on this level could not be included in research assessments of the individual survivors; however, schools were targeted in qualitative research projects, both regarding how they coped with individual survivors and with the pupils in general.

On *the societal level*, the general Norwegian population was targeted in a separate study. This population study was timed to match wave one of the interviews with survivors and their parents and included some identical measures. The aim was to do two things simultaneously: 1) to investigate the relationship of proximity to the terror and distress in the general population, and 2) sample and assess comparison groups for both survivors and their parents. Responses in the general population had been examined after four prior terrorist attacks: the Oklahoma City Bombing (Smith, Christiansen, Vincent, & Hann, 1999), the 9/11 terrorist attacks in the United States (Schlenger et al., 2002), the 2004 train attacks in Madrid (Miguel-Tobal et al., 2006), and the 7th July 2005 London bombings (Rubin, Brewin, Greenberg, Simpson, & Wessely, 2005). These studies showed that terrorist attacks can influence safety perceptions, well-being, and mental health in the general population. In the Norwegian population, people retrospectively reported intense emotional responses the first weekend, dominated by a feeling of unreality and sadness (both reported by 80–90%), with 50% reporting that they cried (Thoresen, Aakvaag, Wentzel-Larsen, Dyb, & Hjemdal, 2012). On the day of the attack, more than half reported being worried about the safety of someone close, and one-fourth of our respondents knew a victim. Thus, psychological proximity in the population was high, and psychological proximity was still associated with posttraumatic stress reactions at 4–5 months after the attack. A significant minority (30%) reported still feeling less safe than before the attacks.

In the social–ecological model, the levels interact. For example, social support is dependent in part on relationship and community level factors. What happens to social support when the surroundings are so affected? Based on a Swedish exploration of social support in a 15-year follow-up of survivors from the Estonia ferry disaster (Arnberg, Hultman, Michel, & Lundin, 2013), the research team developed a measure of “social support barriers.” The team documented that Utøya survivors refrained from seeking help and support when they needed it because they felt that other people were tired of hearing about it, other people had enough with their own problems, others would think they were too caught up with what happened, and more (Thoresen, Jensen, Wentzel-Larsen, & Dyb, 2014). Such barriers were highly predictive of later mental health problems in the survivors, much more so than perceived social support.

Ongoing research will include continued efforts to knit together the levels of the social–ecological model. For example, the team plans to investigate how survivors’ long-term development is influenced by parental coping and vice versa. The team will also investigate community factors, including unemployment rate, education level, availability of health services, and more, that is, sociodemographic data for the communities in which survivors and parents live. Overall, the experience of the teams responding to the Utøya attack is that a social–ecological model fits well as an overarching model for designing studies and services following terrorist attacks.

## Challenges and opportunities for trauma professionals

As mentioned above, we hope to have shown how the public health perspective may help to develop prevention approaches as well as extend the reach of early interventions and treatments. However, a public health approach is not without challenges. Mental health issues (and thus trauma-related issues) are largely ignored in the global public health agenda; thus, it is important for trauma professionals and our professional organizations to support public health efforts to address mental health causes in general and to reduce the stigma and discrimination that is often associated with mental health problems. Similarly, there is a need to expand and develop the global mental health workforce so that it is possible to respond rapidly as trauma and disasters develop worldwide. Expanding such a workforce to meet the needs of low and middle income countries is a true challenge. There is also a need for additional research from low and middle income countries, to inform appropriate preventive interventions that may differ from approaches that work in higher income, industrialized countries.

In addition, there are methodological challenges. Researchers accustomed to working with and measuring the individual impact of traumas may need to rethink their methodologies in order to capture societal impacts. Public health–oriented studies are likely to emphasize generalizability and reach as opposed to internal validity (Koepsell, Zatzick, & Rivara, 2011). In fact, epidemiologic sampling frames can be used as randomized clinical experiments. For example, Hearst, Newman, and Hulley (1986) studied men who had been eligible for the US military draft in two US states during the era of the US–Vietnam war (draft status was random, based on day and month of birth). Draft-eligible men had elevated mortality from all causes as well as suicide and motor vehicle accidents. The researchers attributed this to military service (though military service was not directly measured). Similarly, there are a number of studies that examine state policies in the United States regarding firearms and the effects on health. These studies demonstrate that more and stricter policies are associated with lower rates of non-fatal firearm injuries (Simonetti, Rowhani-Rahbar, Mills, Young, & Rivara, 2015) and homicide (Webster & Wintemute, 2015). The distinguishing feature of these studies is that they include the general population as the denominator rather than only those individuals who are trauma exposed or seeking treatment. This population approach is useful for the development and refinement of policies and can also inform clinical prevention studies (Zatzick & Galea, 2007). Particularly informative may be studies that connect interventions across various levels (e.g., the impact of policies on individual outcomes) (Glasgow & Emmons, 2007).

Achieving a public health approach to trauma is not without tensions. For example, there may be clashes between individual freedoms and societal good (as in the case of efforts to achieve gun control in the United States), poverty versus wealth, politics versus science, or physical health versus mental health. As preventive efforts move to the community and societal levels, it may be difficult to measure their effectiveness because data from large populations will be necessary. Nevertheless, because the potential reach of such interventions is vast, it is incumbent on public health trauma researchers to develop appropriate studies.

Professional organizations such as ESTSS and ISTSS can and should play an important role in promoting a public health approach. They should promote the inclusion of trauma in the global public health agenda and include public health in their activities. Professional societies can help to provide data and insights that will guide decision makers to develop effective public health policies to prevent and mitigate the adverse effects of trauma. They should encourage members to address the gaps in international trauma and public health research. They should promote and participate in developing and maintaining a trauma-informed global public health workforce. In addition, they can improve equitable access to effective integrated and trauma-informed approaches to care. Finally, professional organizations can help society to understand trauma and its sequelae, thereby reducing stigma and discrimination associated with trauma and mental health issues.

## References

[CIT0001] Ajduković D (2013). Introducing the notion of social context of collective trauma to ESTSS. European Journal of Psychotraumatology.

[CIT0002] Anda R.F, Brown D.W, Dube S.R, Bremner D, Felitti V.J, Giles W.H (2008). Adverse childhood experiences and chornic obstructive pulmonary disease in adults. American Journal of Preventive Medicine.

[CIT0003] Anda R.F, Butchart A, Felitti V.J, Brown D.W (2010). Building a framework for global surveillance of the public health implications of adverse childhood experiences. American Journal of Preventive Medicine.

[CIT0004] Arnberg F.K, Hultman C.M, Michel P.-O, Lundin T (2013). Fifteen years after a ferry disaster: Clinical interviews and survivors’ self-assessment of their experience. European Journal of Psychotraumatology.

[CIT0005] Breslau N, Davis G.C, Peterson E.L, Schultz L.R (2000). A second look at comorbidity in victims of trauma: The posttraumatic stress disorder–major depression connection. Biological Psychiatry.

[CIT0006] Breslau N, Davis G.C, Schultz L.R (2003). Posttraumatic stress disorder and the incidence of nicotine, alcohol, and other drug disorders in persons who have experienced trauma. Archives of General Psychiatry.

[CIT0008] Brunet A, Bousquet Des Groseilliers I, Cordova M, Ruzek J (2013). Randomized controlled trial of a brief dyadic cognitive–behavioral intervention designed to prevent PTSD. European Journal of Psychotraumatology.

[CIT0009] Costello E.J, Angold A, Cicchetti D, Cohen D (1995). Developmental epidemiology. Developmental psychopathology, Vol 1: Theory and methods.

[CIT0010] Cox C, Kenardy J (2010). A randomised controlled trial of a web-based early intervention for children and their parents following accidental injury. Journal of Pediatric Psychology.

[CIT0011] Dahlberg L.L, Krug E.G, Krug E, Dahlberg L.L, Mercy J.A, Lozano R (2002). Violence: A global public health problem. World report on violence and health.

[CIT0013] Felitti V.J, Anda R.F, Nordenberg D, Williamson D.F, Spitz A.M, Edwards V, Marks J. S (1998). Relationship of childhood abuse and household dystunction to many of the leading causes of death in adults: The Adverse Childhood Experiences (ACE) study. American Journal of Preventive Medicine.

[CIT0073] Foss A. B, Østli K (2011). Vi er jo frelserne, sier han, vi skal befri Europa fra tyranni.

[CIT0016] Gentilello L.M, Rivara F.P, Donovan D.M, Jurkovich G.J, Daranciang E, Dunn C.W, Ries R.R (1999). Alcohol interventions in a trauma center as a means of reducing the risk of injury recurrence. Annals of Surgery.

[CIT0017] Glasgow R.E, Emmons K.M (2007). How can we increase translation of research into practice? Types of evidence needed. Annual Review of Public Health.

[CIT0018] Goldberg J, Magruder K.M, Forsberg C.W, Kazis L.E, Ustün T.B, Friedman M.J, Smith N.L (2014). The association of PTSD with physical and mental health functioning and disability (VA Cooperative Study #569: The course and consequences of posttraumatic stress disorder in Vietnam-era Veteran twins). Quality of Life Research.

[CIT0072] Gordon R.S (1983). An operational classification of disease prevention. Public Health Reports.

[CIT0019] Guha-Sapir D, Hoyois P, Below R (2014). Annual disaster statistical review 2013: The numbers and trends.

[CIT0020] Hearst H, Newman T.B, Hulley S.B (1986). Delayed effects of the military draft on mortality. New England Journal of Medicine.

[CIT0021] Hinde J.M, Bray J.W, Aldridge A, Zarkin G.A (2015). The impact of a mandated trauma center alcohol intervention on readmission and cost per readmission in Arizona. Medical Care.

[CIT0022] Hoffman B (1999). The mind of the terrorist: Perspectives from social psychology. Psychiatric Annals.

[CIT0023] Kassam-Adams N, Marsac M.L, Kohser K, Kenardy J, March S, Winston F.K (2016). Pilot randomized controlled trial of a novel web-based intervention to prevent posttraumatic stress in children following medical events. Journal of Pediatric Psychology.

[CIT0024] Kawachi I, Subramanian S (2006). Measuring and modeling the social and geographic context of trauma: A multilevel modeling approach. Journal of Trauma Stress.

[CIT0025] Kazdin A, Blase S (2011). Rebooting psychotherapy research and practice to reduce the burden of mental illness. Perspectives on Psychological Science.

[CIT0026] Kessler R.C, Üstün B (2008). The WHO world mental health surveys. Global perspectives of mental health surveys.

[CIT0028] Koepsell T.D, Zatzick D.F, Rivara F.P (2011). Estimating the population impact of preventive interventions from randomized trials. American Journal of Preventive Medicine.

[CIT0029] Laborde D.J, Magruder K.M, Caye J, Parrish T (2013). Feasibility of disaster mental health preparedness training for black communities. Disaster Medicine and Public Health Preparedness.

[CIT0032] Magruder K.M, Frueh B.C, Knapp R.G, Davis L, Hamner M.B, Martin R.H, Arana GW (2005). Prevalence of posttraumatic stress disorder in VA primary care clinics. General Hospital Psychiatry.

[CIT0033] Magruder K.M, Serpi T, Kimerling R, Kilbourne A.M, Collins J.F, Cypel Y, Kang H (2015). Prevalence of post-traumatic stress disorder in Vietnam-era women veterans: The Health ViEWs Study. JAMA-Psychiatry.

[CIT0034] Marsac M.L, Kassam-Adams N, Hildenbrand A.K, Kohser K, Winston F.K (2011). After the injury: Initial evaluation of a web-based intervention for parents of injured children. Health Education Research.

[CIT0035] McLaughlin K.A, Green J.G, Gruber M.J, Sampson N.A, Zaslavsky A, Kessler R.C (2012). Childhood adversities and first onset of psychiatric disorders in a national sample of adolescents. Archives of General Psychiatry.

[CIT0036] Miguel-Tobal J.J, Cano-Vindel A, Gonzalez-Ordi H, Iruarrizaga I, Rudenstine S, Vlahov D, Galea S (2006). PTSD and depression after the Madrid March 11 train bombings. Journal of Traumatic Stress.

[CIT0037] Mouthaan J, Sijbrandij M, De Vries G.-J, Reitsma J.B, Van De Schoot R, Goslings J.C, Olff M (2013). Internet-based early intervention to prevent posttraumatic stress disorder in injury patients: Randomized controlled trial. Journal of Medical Internet Research.

[CIT0038] Mouthaan J, Sijbrandij M, Reitsma J.B, Gersons B.P.R, Olff M (2014). Comparing screening instruments to predict posttraumatic stress disorder. PLoS One.

[CIT0039] Muñoz R.F, Bunge E.L, Chen K, Schueller S.M, Bravin J.I, Shaughnessy E.A, Pérez-Stable E.J (2015). Massive open online interventions: A novel model for delivering behavioral-health services worldwide. Clinical Psychological Science.

[CIT0040] Nakagawa Y, Shaw R (2004). Social capital: A missing link in disaster recovery. International Journal of Mass Emergencies and Disasters.

[CIT0041] Olff M (2015). Mobile mental health: A challenging research agenda. European Journal Of Psychotraumatology.

[CIT0042] Olff M, Langeland W, Draijer N, Gersons B.P.R (2007). Gender differences in posttraumatic stress disorder. Psychological Bulletin.

[CIT0043] Olff M, Van Zuiden M, Bakker A (2015). Early interventions: From e-health to neurobiology. European Journal of Psychotraumatology.

[CIT0045] Price M, Ruggiero K.J, Ferguson P.L, Patel S.K, Treiber F, Couillard D, Fahkry S.M (2014). A feasibility pilot study on the use of text messages to track PTSD symptoms after a traumatic injury. General Hospital Psychiatry.

[CIT0046] Qureshi S.U, Kimbrell T, Pyne J.M, Magruder K.M, Hudson T.J, Petersen N.J, Kunik M.E (2010). Greater prevalence and incidence of dementia in older veterans with PTSD. Journal of the American Geriatrics Society.

[CIT0047] Qureshi S.U, Pyne J.M, Magruder K.M, Schulz P.E, Kunik M.E (2009). The link between post-traumatic stress disorder and physical comorbidities: A systematic review. Psychiatric Quarterly.

[CIT0048] Roberts A.L, Austin S.B, Corliss H.L, Vandermorris A.K, Koenen K.C (2010). Pervasive trauma exposure among US sexual orientation minority adults and risk of posttraumatic stress disorder. American Journal of Public Health.

[CIT0050] Rothbaum B.O, Kearns M.C, Price M, Malcoun E, Davis M, Ressler K.J, Houry D (2012). Early intervention may prevent the development of posttraumatic stress disorder: A randomized pilot civilian study with modified prolonged exposure. Biological Psychiatry.

[CIT0051] Rubin G.J, Brewin C.R, Greenberg N, Simpson J, Wessely S (2005). Psychological and behavioural reactions to the bombings in London on 7 July 2005: Cross sectional survey of a representative sample of Londoners. BMJ.

[CIT0052] Ruggiero K.J, Price M, Adams Z, Stauffacher K, McCauley J, Danielson C, Resnick H.S (2015). Web intervention for adolescents affected by disaster: Population-based randomized controlled trial. Journal of the American Academy of Child & Adolescent Psychiatry.

[CIT0053] Schlenger W.E, Caddell J.M, Ebert L, Jordan B.K, Rourke K.M, Wilson D, Kulka R.A (2002). Psychological reactions to terrorist attacks: Findings from the National Study of Americans’ Reactions to September 11. JAMA.

[CIT0054] Scott K.M, Koenen K.C, Aguilar-Gaxiola S, Alonso J, Angermeyer M.C, Benjet C, Kessler R.C (2013). Associations between lifetime traumatic events and subsequent chronic physical conditions: A cross-national, cross-sectonal study. PLoS One.

[CIT0055] Sijbrandij M, Reitsma J.B, Roberts N.P, Engelhard I.M, Olff M, Sonneveld L, Bisson J.I (2013). Self-report screening instruments for post-traumatic stress disorder (PTSD) in survivors of traumatic experiences. Cochrane Database of Systematic Reviews.

[CIT0056] Simonetti J.A, Rowhani-Rahbar A, Mills B, Young B, Rivara F.P (2015). State firearm legislation and nonfatal firearm injuries. American Journal of Public Health.

[CIT0057] Smith D.W, Christiansen E.H, Vincent R, Hann N.E (1999). Population effects of the bombing of Oklahoma City. The Journal of the Oklahoma State Medical Association.

[CIT0058] Sprague S, Olff M (2014). Intimate partner violence and mental health. European Journal of Psychotraumatology.

[CIT0059] Stoltenborgh M, Bakermans-Kranenburg M.J, Van Ijzendoorn M.H, Alink L.R (2013). Cultural–geographical differences in the occurrence of child physical abuse? A meta-analysis of global prevalence. International Journal of Psychology.

[CIT0060] Stoltenborgh M, Van Ijzendoorn M.H, Euser E.M, Bakermans-Kranenburg M.J (2011). A global perspective on child sexual abuse: Meta-analysis of prevalence around the world. Child Maltreatment.

[CIT0061] Strøm I.F, Thoresen S, Wentzel-Larsen T, Sagatun Å, Dyb G (2014). A prospective study of the potential moderating role of social support in preventing marginalization among individuals exposed to bullying and abuse in junior high school. Journal of Youth and Adolescence.

[CIT0062] Substance Abuse and Mental Health Services Administration (2014). SAMHSA's concept of trauma and guidance for a trauma-informed approach.

[CIT0063] Thoresen S, Aakvaag H.F, Wentzel-Larsen T, Dyb G, Hjemdal O.K (2012). The day Norway cried: Proximity and distress in Norwegian citizens following the 22nd July 2011 terrorist attacks in Oslo and on Utøya Island. European Journal of Psychotraumatology.

[CIT0064] Thoresen S, Jensen T.K, Wentzel-Larsen T, Dyb G (2014). Social support barriers and mental health in terrorist attack survivors. Journal of Affective Disorders.

[CIT0065] Turner (2015). Refugee Blues: A UK and European perspective. European Journal of Psychotraumatology.

[CIT0066] United Nations Children's Fund (2014). Hidden in plain sight: A statistical analysis of violence against children.

[CIT0067] Webster D.W, Wintemute G.J (2015). Effects of policies designed to keep firearms from high-risk individuals. Annual Review of Public Health.

[CIT0070] Zatzick D.F, Galea S (2007). An epidemiologic approach to the development of early trauma focused intervention. Journal of Traumatic Stress.

[CIT0071] Zwillich T (2006). Health care remains basic in New Orleans. Lancet.

